# Chinese Medicines for Preventing and Treating Radiation-Induced Pulmonary Injury: Still a Long Way to Go

**DOI:** 10.3389/fphar.2019.00927

**Published:** 2019-09-05

**Authors:** Yan Ding, Yuechao Liu, Hongliang Li, Yong Li, Minglun Li, Ming Liu, Xianhe Wang, Fengjun Cao, Xuanbin Wang

**Affiliations:** ^1^Laboratory of Chinese Herbal Pharmacology, Oncology Center, Renmin Hospital, Hubei University of Medicine, Shiyan, China; ^2^Department of Radiation Oncology, University Hospital, LMU, Munich, Germany; ^3^Biomedical Research Institute, Hubei Key Laboratory of Wudang Local Chinese Medicine Research, Hubei University of Medicine, Shiyan, China

**Keywords:** Chinese medicines, radiation-induced pulmonary injury, prevention, treatment, tonics, review

## Abstract

Thoracic radiotherapy is a mainstay of the treatment for lung, esophageal, and breast cancers. Radiation-induced pulmonary injury (RIPI) is a common side effect of thoracic radiotherapy, which may limit the radiotherapy dose and compromise the treatment results. However, the current strategies for RIPI are not satisfactory and may induce other side effects. Chinese medicines (CMs) have been used for more than a thousand years to treat a wide range of diseases, including lung disorders. In this review, we screened the literature from 2007 to 2017 in different online databases, including China National Knowledge Infrastructure (CNKI), Chongqing VIP, Wanfang, and PubMed; summarized the effectiveness of CMs in preventing and treating RIPI; explored the most frequently used drugs; and aimed to provide insights into potential CMs for RIPI. Altogether, CMs attenuated the risk of RIPI with an occurrence rate of 11.37% *vs.* 27.78% (*P* < 0.001) compared with the control groups. We also found that CMs (alone and combined with Western medical treatment) for treating RIPI exerted a higher efficacy rate than that of the control groups (78.33% *vs.* 28.09%, *P* < 0.001). In the screened literature, 38 CMs were used for the prevention and treatment of RIPI. The top five most frequently used CMs were Astragali Radix (with a frequency of 8.47%), Ophiopogonis Radix (with a frequency of 6.78%), Glycyrrhizae Radix et Rhizome (with a frequency of 5.08%), Paeoniae Radix Rubra (with a frequency of 5.08%), and Prunellae Spica (with a frequency of 5.08%). However, further high-quality investigations in CM source, pharmacological effects and underlying mechanisms, toxicological aspects, and ethical issues are warranted. Taken together, CMs might have a potential role in RIPI prevention and treatment and still have a long way to investigate.

## Introduction

Thoracic radiotherapy is a mainstay of treatment for patients who have cancer, e.g., lung cancer, esophageal cancer, and breast cancer. Radiation-induced pulmonary injury (RIPI) is a common side effect, including two phases, acute radiation pneumonitis and chronic radiation-induced pulmonary fibrosis (Cao et al., 2017; Huang et al., 2017). The symptoms include a dry cough, shortness of breath, chest pain, fever, and even severe respiratory failure and death (Huang et al., 2017). RIPI is highly likely to occur with a high dose and dose rate of radiation. Capillary endothelial and type I cells (epithelial lining cells) appear to be most susceptible to RIPI (Abid et al., 2001). RIPI may lead to a reduction and damage in pulmonary function, which involves a cascade of inflammatory events, including oxidative damage (Demirel et al., 2016), sphingomyelin hydrolysis, and apoptotic DNA degradation (Abid et al., 2001). Patients with RIPI typically suffer from dyspnea with a decreased vital capacity (VC), forced vital capacity (FVC), forced expiratory volume in 1 s (FEV1), alveolar volume (V_A_), transfer factor of carbon monoxide (T_L, CO_), and residual volume (RV) (Enache et al., 2013). On the cellular and molecular level, radiation may result in damage to the alveolar epithelial cells as well as vascular endothelial cells and the secretion of a large number of pro-inflammatory and pro-fibrotic cytokines, including transforming growth factor (TGF)-β1, interleukin-13 (IL-13), endothelin-1 (ET-1), platelet-derived growth factor (PDGF), cyclooxygenase (COX), and prostaglandin E2 (PGE2). Furthermore, type 1 helper T cells (Th1 cells) may affect RIPI by secreting IL-4 and IL-13, while type 2 helper T cells (Th2 cells) induce collagen synthesis for tissue remodeling and fibrosis (Huang et al., 2017). To date, the treatment strategies for RIPI are mainly based on glucocorticoids, angiotensin-converting enzyme inhibitors (ACEIs), and pentoxyphylline (Deng et al., 2017). Thus, a comprehensive review of the effects of CMs on RIPI may help discover the most effective CMs. In this review, studies of CMs in RIPI were retrieved from online databases from 2007 to 2017. The literature was screened using the inclusive and exclusive criteria and analyzed using statistical software.

## Materials and Methods

### Data Retrieval and Collection

The keywords “radiation-induced pulmonary/lung injury, “radiation-induced pneumonitis/pulmonary fibrosis, and “Chinese medicine/ traditional Chinese medicine/ Chinese herbal medicine” were used to retrieve studies of CMs in RIPI from 2007 to 2017 from the online databases of China National Knowledge Infrastructure (CNKI), Chongqing VIP, Wangfang, and PubMed. The duplicates were discarded. The overall efficacy, changes of pulmonary function, underlying mechanisms, CM species and families, traditional use, and usage frequency in these studies were summarized and analyzed. The study was approved by the Ethical Commitee of Hubei University of Medicine.

### Criteria of Inclusion

The inclusion criteria were as follows:

- The contents of the literature involved the clinical effects of CMs on RIPI, including radiation-induced pneumonitis and/or pulmonary fibrosis.
- The references included pure compounds, fractions, and formulae of CMs. The CMs were composed of fungi, plants, animals, and their parts, and minerals.
- The design of the studies was randomized and controlled.
- The studies described some parameter of lung function.

### Criteria of Exclusion

The data from the following literature were excluded:

- The literature was associated with neither CMs nor RIPI.
- The pure compounds were not naturally from CMs but were chemical derivatives.
- The species of CMs were not given or could not be determined.
- The fractions and/or formulae of CMs were described without the extraction methodology.
- The components of the formulae were not given.
- The manufacturer and/or manufacturing data were not stated if marketed CM preparations were used.
- The dosage of the drugs was not given.
- Only the overall efficacy of CMs on RIPI was described without any assessment of lung function or biochemical indices.
- The studies were not randomized and controlled.
- The literature did not claim any ethical approvals, and the studies without desclaim of patients’ agreement or signing informed consents.
- The study was any review or a meta-analysis.

### Statistical Analysis

The data were selected based on the criteria defined in the inclusion and exclusion criteria for clinical settings, and they were summarized and analyzed using SPSS19.0 (SPSS Inc., Chicago, IL, USA) and GraphPad Prism 5.0 (GraphPad Software, Inc. La Jolla, CA, USA). Data were presented as the mean ± standard deviation (SD). One-way ANOVA was performed for multiple comparisons analysis, and two-sided Student’s *t* test was used to compare differences between the two groups. *P* < 0.05 was considered statistically significant.

## Results

### General Information on Literature Retrieval

**Figure 1:**
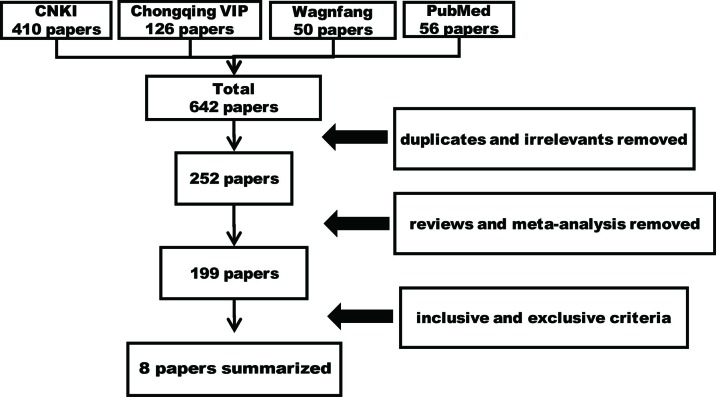
The workflow of retrieving literature.

**Figure 2:**
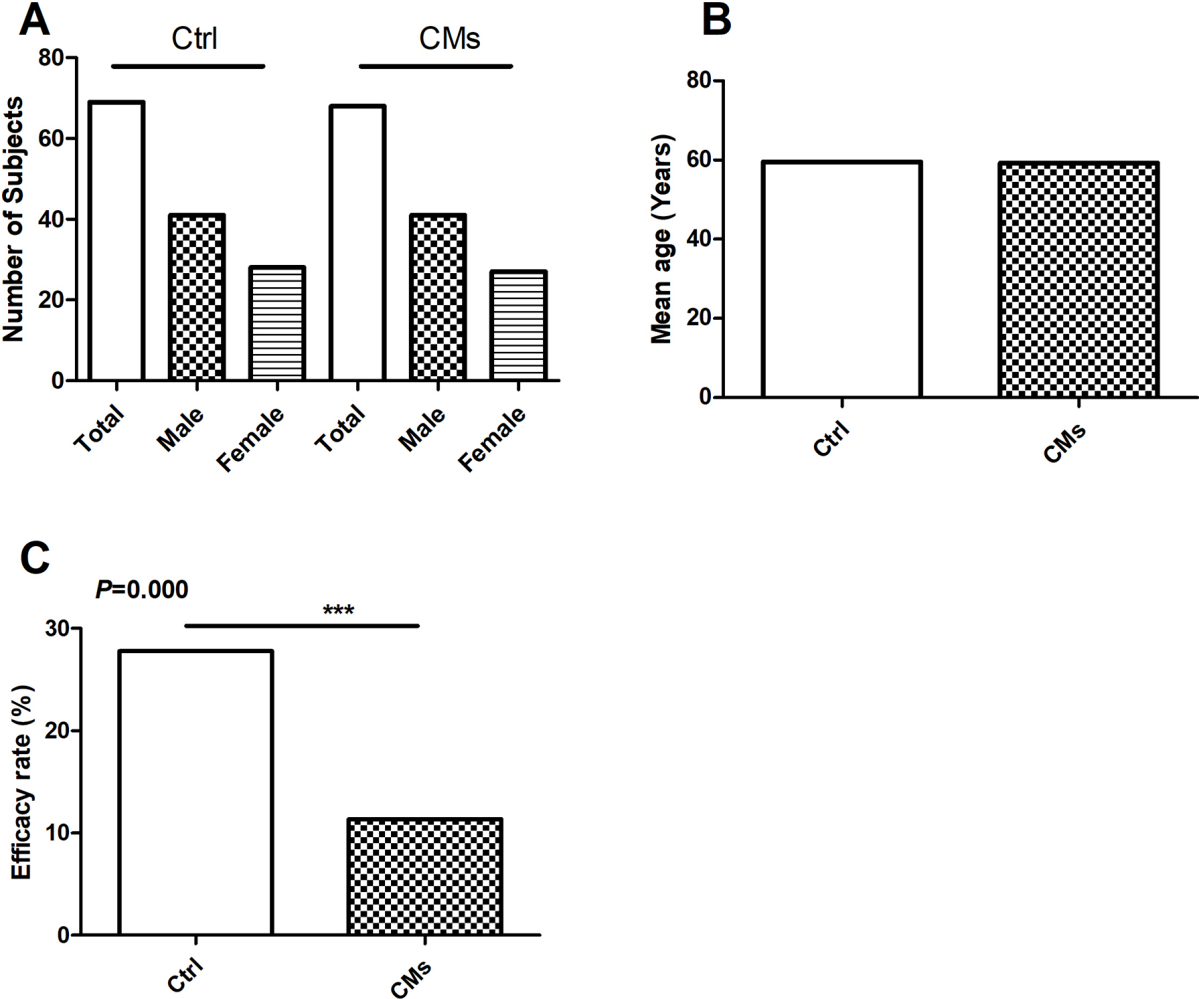
Efficacy of Chinese Medicines (CMs) for preventing radiation-induced pulmonary injury (RIPI) in patients in the clinical studies. **(A)** The number and gender of patient comparison between the control and the CM-treated groups. **(B)** The average age of patient comparison between the control and the CM-treated groups. **(C)** The RIPI occurrence rate of the combination treatment of CMs with Western medical strategies *vs.* the Western medical strategies alone (Ctrl). ***P < 0.001.

**Figure 3:**
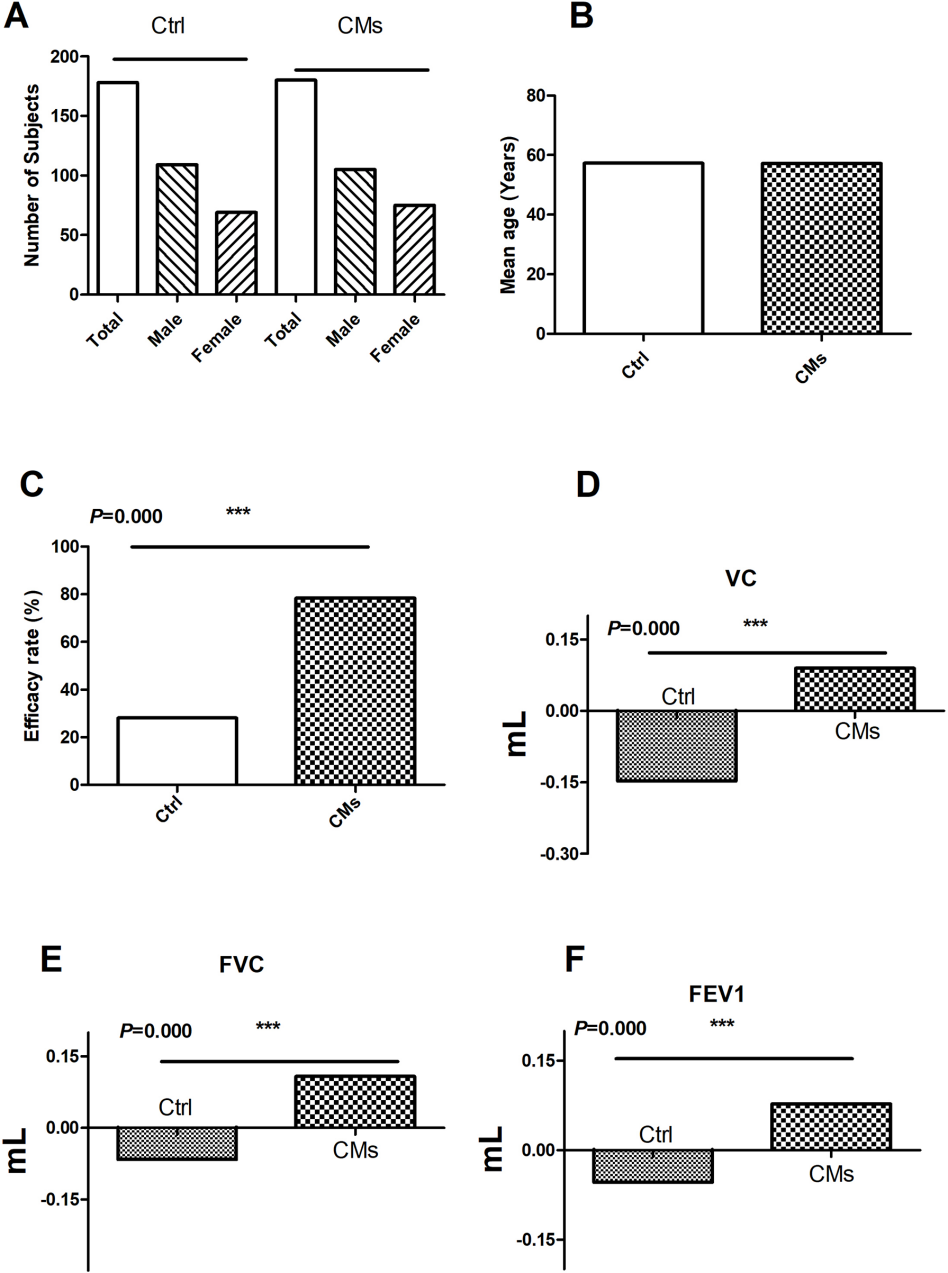
Efficacy of CMs for treating RIPI patients in the clinical studies. **(A)** The number and gender of patient comparison between the control and the CM-treated groups. **(B)** The age of patient comparison between the control and the CM-treated groups. **(C)** Overall efficacy comparison between the control and the CM-treated groups. **(D)** The comparison of VC changes before and after CM treatment. **(E)** The comparison of FVC changes before and after CM treatment. **(F)** The comparison of FEV1 changes before and after CM treatment. Ctrl: the Western medical strategies alone; CMs: the CMs or CMs combined with Western medical strategies. ***P < 0.001.

A total of 642 papers were retrieved, including 390 duplicates and irrelevant literature, 53 reviews and meta-analysis, and 191 articles that were excluded using the selection criteria. As a result, eight studies that met the selection criteria were summarized and analyzed (**Figure 1**). Ethics committees approved all clinical trials, and all patients agreed on participating studies and signed informed consents. The CMs examined in eight studies (five for prevention, one for treatment, and two for both) included eight formulae (**Table 1**). Furthermore, some CMs were used with a high frequency (**Table 2**). In these papers, the 883 patients were randomly divided into two groups, the control group (Ctrl, 407 patients), the CM-preventing group (CMs, 343 patients) (**Figure 2**), and the CM-treating group (CMs, 180 patients) (**Figure 3**).

**Table 1:** List of CMs formulae for RIPI.

Name	Main components	Dose	Effect and mechanism	Stage	References
Qing Zao Jiu Fei Decoction-1	Mori folium (9 g), Gypsum fibrosum (8 g), Glycyrrhizae radix et Rhizoma (3 g), Sesame (3 g), Asini corii colla (3 g), Eriobotryae folium (3 g), Ginseng radix et Rhizoma (2 g), Armeniacae Semen Amarum (2 g), Ophiopogonis radix (4 g), Astragali radix (3 g)	100 ml	TGF-β1 ↓; IL-1 ↓	RIPI	(Xia et al., 2010)
Qing Re Huo Xue San Jie Fang	Prunella spica (15 g), Cremastrae Pseudobulbs Pleiones Pseudobulbus (15 g), Forsythiae Fructus (12 g), Astragali radixn (125 g), Paeoniae radix rubra (10 g), Moutan cortex (10 g)	100 ml	TNF-α ↓; IL-1 ↓; TGF-β1 ↓; MMP-9 ↓	RIPI	(Tu et al., 2015)
Bu Qi Yang Yin Fang	Rhodiola crenulate radix et Rhizoma (20 g), Astragali radix (20 g), Lycii Fructus (15 g), Pseudostellariae radix (15 g), Ophiopogonis radix (15 g), Eriobotryae folium (15 g), Angelicae Sinensis Radix (15 g), Prunellae spica (10 g), Gypsum fibrosum (10 g), Curcumae radix (10 g), Curcumae longae rhizoma (10 g)	100 ml	VC ↑; FVC ↑; FEV1 ↑; TGF-β1 ↓; TNF-α ↓	RIPI	(Zhou and Chen, 2017)
Qi Shen Yi Fei Decoction	Astragali Radix (30 g), Codonopsis Radix (15 g), Ophiopogonis Radix (15 g), Prunellae spica (15 g), Fritillariae cirrhosia bulbus (12g), Forsythiae Fructus (12 g), Astragali Radix (12 g), Descurainiae Semen lepidii Semen (10 g), Persicae semen (10 g), Moutan Cortex (10 g), Salviae Miltiorrhizae Radix et Rhizoma (10 g), Paeoniae Radix Rubra (10 g), Glycyrrhizae Radix et Rhizoma (6 g)	100 ml	VC ↑; FVC ↑; FEV1 ↑; IL-6 ↓; TNF-α ↓; TGF-β1 ↓	RP	(Shi et al., 2016)
Qing Fei Decoction	Rehmanniae Radix (12 g), Ophiopogonis Radix (9 g), Scrophulariae Radix (9 g), Sheng Glycyrrhizae Radix et Rhizoma (3 g), Menthae Haplocalycis Herba (3 g), Asteris Radix et Rhizoma (5 g), Moutan Cortex (5 g), Paeoniae Radix Alba (5 g)	100 ml	IL-1 ↓; TNF-α ↓	RIPI	(Li et al., 2017)
Wu Wei Xiao Du Yin Jia Jian Fang	Lonicera Japonicae Flos (20 g), Viola Herba (15 g), Ford Nervilia Herb or Tuber (15 g), Taraxaci Herba (15 g), Chrysanthemi Indici Flos (15 g), Pseudostellariae Radix (6 g), Schisandrae Chinensis Fructus (6 g)	100 ml	TGF-β1 ↓	RP	(Chen et al., 2017)
Xue Bi Jing Injection	Carthami Flos, Paeoniae Radix Rubra, Chuanxiong Rhizoma, Salviae Miltiorrhizae Radix et Rhizoma, Angelicae Sinensis Radix	100 ml	HMGB1 ↓; TGF-β1 ↓; IL-6 ↓; TNF-α ↓	RIPI	(Tan et al., 2014)
Compound Ku Shen Injection	Sophorae Flavescentis Radix, Smilacis Glabrate Rhizoma	30 ml	TGF–β1 ↓; fibronectin ↓; TNF-α ↓; CTGF ↓	RIPI RP	(Luo et al., 2015)

CTGF, connective tissue growth factor; EMT, epithelial–mesenchymal transition; ERK, extracellular signal-regulated (protein) kinase; ET-1, endothelin-1; FEV1, forced expiratory volume in 1 s; FVC, forced vital capacity; GSK, glycogen synthase kinase; HMGB1, high mobility group protein B 1; HP, hydroxyproline; IFN-*γ*, interferon-*γ*; IL-1, interleukin-1; IL-10, interleukin-10; IL-4, interleukin-4; IL-6, interleukin-6; LDH, lactate dehydrogenase; MDA, malondialdehyde; MMP, matrix metalloproteinase-9; RIPF, radiation-induced pulmonary fibrosis; RIPI, radiation-induced pulmonary injury; ROS, reactive oxygen species; RP, radiation pneumonitis; SOD, superoxide dismutase; SP-A, surfactant protein a; TGF-*β*1, transforming growth factor; TIMP-1, tissue inhibitor of metalloproteinase-1; TNF-*α*, tumor necrosis factor-*α*; VC, vital capacity; NA, not available; *↑*, up-regulation or activation; *↓*, down-regulation or inactivation.

**Table 2:** Species list of CMs for anti-RIPI.

Chinese name	English name	Latin name in the Chinese Pharmacopeia (Version 2015)	Accepted/Synonym name in the Plant List	Frequency (%)	Potential toxicity
黄芪	Astragali Radix	*Astragalus membranaceus* (Fisch). Bge. var. mongholicus (Bge). Hsiao	*Astragalus trimestris* L.	8.47	
		*Astragalus membranaceus* (Fisch). Bge.	*Astragalus mongholicus* Bunge		
麦冬	Ophiopogonis Radix	*Ophiopogon japonicus* (L. f) Ker-GawL	*Ophiopogon japonicus* (Thunb). Ker Gawl.	6.78	
甘草	Glycyrrhizae Radix et Rhizoma	*Glycyrrhiza uralensis* Fisch.	*Glycyrrhiza uralensis* Fisch.	5.08	Hypertension and hypokalemic-induced secondary disorders (Nazari et al., 2017)
		*Glycyrrhiza inflat*e Bat.	*Glycyrrhiza inflat*e Batalin		
		*Glycyrrhiza glabra* L.	*Glycyrrhiza glabra* L.		
赤芍	Paeoniae Radix Rubra	*Paeonia lactiflora* PalL	*Paeonia lactiflora* Pall.	5.08	
		*Paeonia veitchii* Lynch	*Paeonia veitchii* Lynch		
夏枯草	Prunellae spica	*Prunella vulgaris* L.	*Prunella vulgaris* L.	5.08	
当归	Angelicae Sinensis Radix	*Angelica sinensis* (Oliv). Diels	*Angelica sinensis (Oliv).* Diels	3.39	
枇杷叶	Eriobotrya folium	*Eriobotrya japonica* (Thunb). Lindl.	*Eriobotrya japonica* (Thunb). Lindl.	3.39	
连翘	Forsythiae Fructus	*Forsythia suspensa* (Thunb). V ahl	*Forsythia suspensa* (Thunb). Vahl	3.39	
石膏	^a^ Gypsum (CaSO_4_ • 2 H_2_O)		NA	3.39	
金银花	Lonicerae Japonicae Flos	*Lonicera japonica* Thunb.	*Lonicera japonica* Thunb.	3.39	
牡丹皮	Moutan cortex	*Paeonia suffruticosa* Andr.	*Paeonia* × *suffruticosa* Andrews	3.39	
太子参	Pseudostellariae Radix	*Pseudostellaria heterophylla* (Miq). Pax ex Pax et Hoffm.	*Pseudostellaria heterophylla* (Miq). Pax	3.39	
丹参	Salviae Miltiorrhizae Radix et Rhizoma	*Salvia miltiorrhiza* Bge.	*Salvia miltiorrhiza* Bunge	3.39	
苦杏仁	Armeniacae semen amarum	*Prunus armeniaca* L. var. ansu Maxim.	*Prunus armeniaca* L.	1.69	^#^Nausea, vomiting, diarrhea, dizziness, weakness, mental confusion, and convulsions followed by terminal coma and literally death (Chaouali et al., 2013)
		Prunus sibirica L.	*Prunus sibirica* L.		
		*Prunus mandshurica* (Maxim). Koehne	*Prunus mandshurica* (Maxim). Koehne		
		*Prunus armeniaca* L.	*Prunus mume* (Siebold) Siebold & Zucc.		
阿胶	^b^ Asini corii colla	*Equus asinm* L.	NA	1.69	
紫菀	Asteris Radix et Rhizoma	*Aster tataricus* L. f.	*Aster tataricus* L.f.	1.69	
红花	Carthami Flos	*Carthamus tinctorius* L.	*Carthamus tinctorius* L.	1.69	
野菊花	Chrysanthemi indici flos	*Chrysanthemum indicum* L.	*Chrysanthemum indicum* L.	1.69	
川芎	Chuanxiong Rhizoma	*Ligusticum chuanxiong* Hort.	Ligusticum striatum DC.	1.69	
党参	Codonopsis Radix	*Codonopsis pilosula* (Franch). Nannf.	*Codonopsis pilosula* (Franch). Nannf.	1.69	
		*Codonopsis pilosula* Nannf. var. modesta (Nannf). L. T. Shen	*Codonopsis pilosula* (Franch). Nannf.		
		*Codonopsis tangshen* Oliv.	*Codonopsis tangshen* Oliv.		
山慈菇	Cremastrae Pseudobulbs Pleiones Pseudobulbus	*Cremastra appendiculata* (D. Don) *Makino*	Cremastra appendiculata (D. Don) Makino	1.69	
		*Pleione bulbocodioides* (Franch). Rolfe	*Pleione bulbocodioides* (Franch). Rolfe		
		*Pleione yunnanensis* Rolfe	*Pleione yunnanensis* (Rolfe) Rolfe		
姜黄	Curcumae Longae rhizoma	*Curcuma longa* L.	*Curcuma longa* L.	1.69	
郁金	Curcumae radix	*Curcuma wenyujin* Y. H. Chen et C. Ling	*Curcuma aromatica* Salisb.	1.69	
		*Curcuma longa* L.	*Curcuma longa* L.		
		*Curcuma kwangsiensis* S. G. Lee et C. F. Liang	*Curcuma kwangsiensis* S.G.Lee & C.F.Liang		
		*Curcuma phaeocaulis* Val.	*Curcuma phaeocaulis* Valeton		
葶苈子	Descurainiae semen lepidii semen	*Descurainia sophia* (L). Webb. ex Prantl.	*Descurainia sophia* (L). Webb ex Prantl	1.69	
		*Lepidium apetalum* Wild	*Lepidium apetalum* Wild.		
青天葵	Ford Nervilia Herb or Tuber	*Nervilia fordii* (Hance) Schltr.	*Nervilia fordii* (Hance) Schltr.	1.69	
川贝母	Fritillariae cirrhosae bulbus	*Fritillaria cirrhosa* D. Don	*Fritillaria cirrhosa* D. Don	1.69	
		*Fritillaria unibracteata* Hsiao et K. C. Hsia	*Fritillaria unibracteata* P.K.Hsiao & K.C.Hsia		
		*Fritillaria przewalskii* Maxim.	*Fritillaria przewalskii* Maxim. ex Batalin		
		*Fritillaria delavayi* Franch.	*Fritillaria delavayi* Franch.		
		*Fritillaria taipaiensis* P. Y. Li	*Fritillaria taipaiensis* P.Y.Li		
		*Fritillaria unibracteata* Hsiao et K. C. Hsia var wabuensis (S. Y. Tang et S. C. Yue) Z. D. Liu, S. Wang et S. C. Chen	*Fritillaria unibracteata* P.K.Hsiao & K.C.Hsia		
人参	Ginseng Radix et Rhizoma	*Panax ginseng* C. A. Mey.	*Panax ginseng* C.A.Mey.	1.69	Potentiates imatinib-induced hepatotoxicity (Bilgi et al., 2010)
枸杞子	Lycii Fructus	*Lycium barbarum* L.	*Lycium barbarum* L.	1.69	
薄荷	Menthae Haplocalycis Herba	*Mentha haplocalyx* Briq.	*Mentha canadensis* L.	1.69	
桑叶	Mori folium	*Morus alba* L.	*Morus alba* L.	1.69	
白芍	Paeoniae radix alba	*Paeonia lactiflora* Pall.	*Paeonia lactiflora* Pall.	1.69	
桃仁	Persicae semen	*Prunus persica* (L). Batsch	*Prunus persica* (L). Batsch	1.69	
		*Prunus davidiana* (Carr). Franch.	*Prunus davidiana* (Carrière) Franch.		
熟地黄	Rehmanniae Radix Praeparata	*Rehmannia glutinosa* Libosch.	*Rehmannia glutinosa* (Gaertn). DC.	1.69	
红景天	Rhodiola Crenulatae Radix et Rhizoma	*Rhodiola crenulata* (Hook. f. et Thoms). H. Ohba	*Sedum crenulatum* Hook.f. & Thomson	1.69	
五味子	Schisandrae Chinensis Fructus	*Schisandra chinensis* (Turcz). Baill	*Schisandra chinensis* (Turcz). Baill.	1.69	
玄参	Scrophulariae radix	*Scrophularia ningpoensis* Hemsl	*Scrophularia ningpoensis* Hemsl.	1.69	
芝麻	Sesame	*Sesamum indicum* L.	*Sesamum indicum* L.	1.69	
蒲公英	Taraxaci herba	*Taraxacum mongolicum* Hand Mazz.	*Taraxacum mongolicum* Hand.-Mazz.	1.69	
		*Taraxacum borealisinense* Kitam.	*Taraxacum sinicum* Kitag.		

^#^The identified toxic Chinese medicines according to the Chinese Pharmacopeia (Version 2015). ^a^Mineral; ^b^Animal parts; NA: not available.

### Effects of CMs on Preventing RIPI

For the seven studies, the Ctrl groups (with a mean age of 59.5 years) were treated with radiotherapy only, while the CM groups received CM pre-intervention (with a mean age of 59.5 years) in addition to radiotherapy. The total RIPI occurrence rates in the Ctrl and the CM groups were 27.78% and 11.37%, respectively (*P* < 0.001), indicating that CMs significantly attenuated the risk of RIPI (**Figure 2**).

### Overall Efficacy of CMs for Treating RIPI

In the three clinical studies with efficacy data, the treatment groups were CMs alone or combined with Western medicine. The control groups were Western medicine. The results showed that CMs alone and CMs combined with Western medical treatment were more effective than control groups (78.33% *vs.* 28.09%) (*P* < 0.001, **Figure 3**).

Furthermore, the change in pulmonary function was investigated in two studies. In total, 120 patients (74 males and 46 females) were treated with CMs or CMs combined with Western medical treatment. Compared with the control groups (Ctrl, 120 patients including 77 males and 43 females), CMs and CMs combined with Western medical treatment were more useful for the improvement of the VC, FVC, and FEV1, indicating that CMs or CMs combined with Western medical treatment were more effective in improving the impaired lung function (**Figure 3**).

### CMs: Commonly Used Families, Traditional Action Classification, and Frequency of Usage

**Figure 4:**
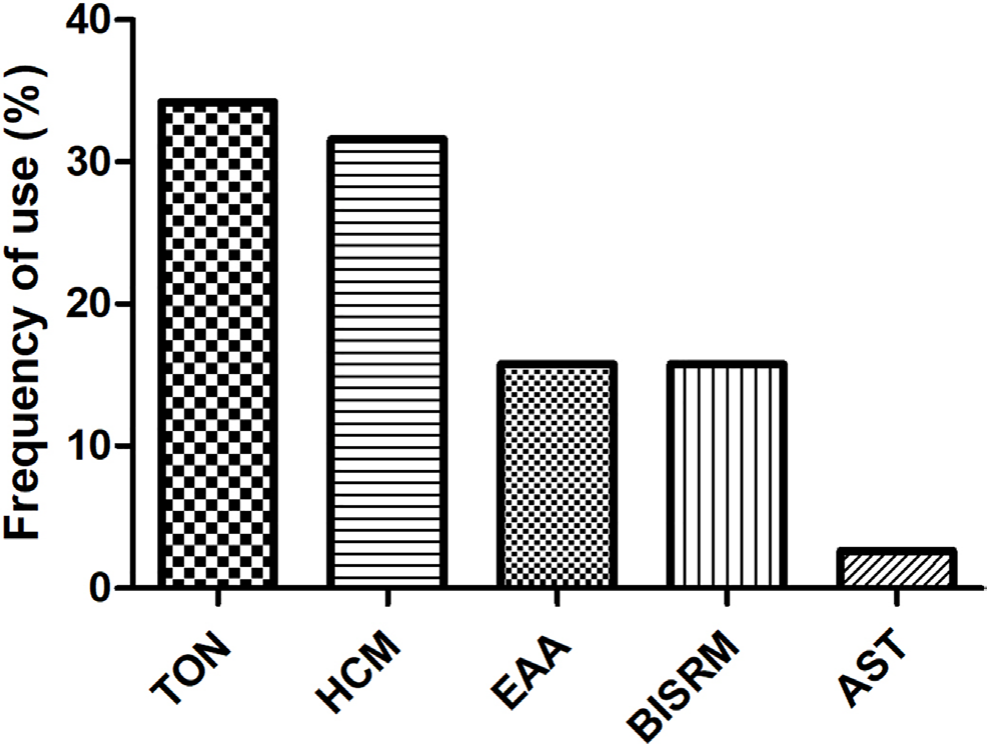
Usage frequency of CMs on RIPI. The usage frequency of traditional action classification of CMs on RIPI. TON, tonics; HCM, heat-clearing medicines; EAA, expectorants, antitussives, and antiasthmatics; BISRM, blood invigorating and stasis resolving medicines AST, astringent medicines.

As CMs for RIPI were used according to their traditional property, it is necessary to explore the relationship between pharmacological effects and traditional function, as well as CM species. We firstly summarized CMs according to traditional action classification (Chen et al., 2017). However, interestingly, only 5 out of 17 current categories of CMs were used. The order according to the frequency from high to low was as follows: tonics (TON, 34.21%); heat-clearing medicines (HCM, 31.58%); expectorants, antitussives, and antiasthmatics (EAA, 15.79%); blood invigorating and stasis resolving medicines (BISRM, 15.79%); and astringent medicines (AST, 2.63%) (**Figure 4**). The top five most frequently used CMs were Astragali radix (with a frequency of 8.47%), Ophiopogonis radix (with a frequency of 6.78%), Glycyrrhizae radix et rhizome (with a frequency of 5.08%), Paeoniae Radix Rubra (with a frequency of 5.08%), and Prunellae spica (with a frequency of 5.08%) (**Table 2**).

## Discussion

Thoracic radiotherapy is the mainstay for the treatment of cancer diseases. However, RIPI is the dose-limiting factor precluding a curative therapy in some cases (Huang et al., 2017). Although several treatment regimens may alleviate RIPI, the therapeutic effects are often unsatisfactory, and these medicines also induce some new side effects (Giridhar et al., 2015). Thus, it is an urgent issue to seek novel drugs for RIPI without serious side effects. CMs have a very long history of curing patients with lung diseases, in which some effective medicines may be found for the prevention and treatment of RIPI (Deng et al., 2017). In this review, we retrieved the most recent publications in the last 10 years, from 2007 to 2017, in different online databases to provide comprehensive insight into the efficacy of CMs on RIPI. We found that eight studies met our criteria. Some CMs were frequently used. In total, eight formulae were used in the reviewed literature. The indices and parameters of pulmonary function were summarized and analyzed to investigate the therapeutic effects of CMs on RIPI quantitatively. The total RIPI occurrence in the CM-preventing groups was significantly lower than that of the control groups (11.37% *vs.* 27.78%) (*P* < 0.001), while the treatment efficacy of CMs was significantly higher than that in the control group (78.33 *vs.* 28.09%, *P* < 0.001). Our results might be helpful to discover the most effective CMs for treating RIPI if the high-quality studies would be conducted.

The underlying mechanisms of CMs on RIPI seem to be complicated. In addition to the well-known inflammatory and fibrotic factors, other factors, such as oxidative response factors and immune cytokines, were also involved. In this review, CMs may be associated with enhancing anti-oxidation, anti-inflammation, and anti-fibrosis properties, and improving immune function. As to the anti-oxidation property, Shen Fu Injection reduced ROS (Bi and Sun, 2015). For anti-inflammation, CMs alleviated RIPI by reducing inflammatory factors such as IL-1, IL-4, IL-6, IL-10, high mobility group protein B1 (HMGB1), prostaglandin (PGE2), and tumor necrosis factor-α (TNF-α) (**Tables 1** and **2**). Regarding enhancing the immune function, Qing Fei Yang Yin Huo Xue Fang activated NK cells. For pulmonary fibrosis, Mai Men Dong Decoction-2 reduced epithelial–mesenchymal transition (EMT) by activation of TGF-β (Liu et al., 2016); Scutellariae Radix for EMT may be associated with its active component, baicalin, which inhibited extracellular regulated protein kinases (ERK)/Glycogen Synthase Kinase 3β(GSK3β) (Lu et al., 2017).

To unveil the relationship between the pharmacological effects and the traditional applications of CMs, the frequency of the species was summarized in **Table 2**, and CM category according to their traditional applications was analyzed in **Figure 4**. All botanical species were checked using the Chinese Pharmacopeia (Version 2015) and the online database of the Plant List (**Table 2**). For the 38 CMs in this review, 36 botanic families, one animal part, and one mineral were used. Interestingly, only five categories of CMs were used for RIPI. In fact, there are up to 17 categories of CMs in use to treat various diseases according to their traditional applications, including exterior-releasing medicines; heat-clearing medicines; medicines that drain downwards; medicines that expel wind and damp; damp-resolving medicines; damp-draining medicines; medicines that warm the interior; Qi-regulating medicines; digestant medicines; medicines that stop bleeding; blood invigorating and stasis resolving medicines; expectorants, antitussives and antiasthmatics; medicines that quiet the spirit; liver-pacifying and wind-extinguishing medicines; resuscitative medicines; tonics; and astringent medicines (Chen, 2017). The reviewed five categories might be closely associated with their pharmacological actions for RIPI. The first class was tonics (e.g., Astragali Radix, Ophiopogonis Radix, and Glycyrrhizae Radix et Rhizoma). Tonics could enhance immune function, which indicates that tonics may attenuate the symptoms by enhancing resistance to RIPI. The second class was heat-clearing medicines (e.g., Paeoniae Radix Rubra and Prunellae Spica). The main pharmacological effects of heat-clearing medicines are anti-inflammation, anti-pathogenic microorganism, adjusting immune function, anti-pyretic effects, and so on. The third class was the expectorants, antitussives, and antiasthmatics (e.g., Eriobotryae Folium, Armeniacae Semen Amarum, and Asteris Radix et Rhizoma), which could dispel phlegm, inhibit coughing, and exert an anti-inflammation effect, and these CMs respond by targeting pneumonitis and pulmonary fibrosis. As for the blood-invigorating and stasis-resolving medicines (e.g., Salviae Miltiorrhizae Radix et Rhizome), it may treat RIPI by anti-oxidative and anti-inflammation (Peng et al., 2019; Yang et al., 2019). The fifth and last class was astringent medicines (Schisandrae chinensis fructus), which could exert cough suppression, anti-fibrosis, and immune regulation. This may be its potential use for RIPI (Chen, 2017; Zhai et al., 2014). Interestingly, the most frequently used species were tonics, Astragali Radix (with a frequency of 8.47%), and Ophiopogonis Radix (with a frequency of 6.78%), indicating that enhancing the immune function is more critical than anti-inflammation in RIPI (**Table 2**).

However, further high-quality investigations should be warranted. The research quality with regards to repeatability and standardization is vital for CMs. The diversity of CM species is a limitation in this review, which may affect the quality of the studies. Although we checked all the CMs with the Chinese Pharmacopeia (Version 2015) and the Plant List (www.theplantlist.org) in this study (**Table 2**), it was still hard to identify the detail species of the used CMs. For the most CMs, there are more than one source for medicinal use. For example, according to the Chinese Pharmacopeia (Version 2015), there are three sources for Glycyrrhizae Radix et Rhizoma, *Glycyrrhiza* uralensis Fisch. (accepted name in the Plant List), *Glycyrrhiza* inflate Bat. (accepted name in the Plant List), and *Glycyrrhiza* glabra L. (accepted name in the Plant List). These three different species may have different components, which might result in different effects. Actually, 11 CMs had more than one species in this review (28.95% out of the total CMs).

Furthermore, because of the similar Chinese names but very different sources, the species of CMs that were not given or could not be determined were excluded in this review. The paper only describing Sha Shen (沙参) was not considered in this review because there are two different resources of Sha Shen, where one is named Bei Sha Shen (Glehniae Radix in English, 北沙参 or North Sha Shen), which is the root of Glehnia littoralis Fr. Schmidt ex Miq. (Family: Umbelliferae) (Committee of Chinese Pharmacopeia, 2015a), while the other is named Nan Sha Shen (Adenophorae Radix in English, 南沙参 or South Sha Shen), which is the root of Adenophora tetraphylla (Thunb). Fisch or Adenophora stricta Miq. (Family: Campanulaceae) (Committee of Chinese Pharmacopeia, 2015b). This means that they share similar Chinese names but are different species, which may result in different pharmacological/toxicological effects. The other similar reason for the standardization of CM research is to verify the process of CM preparation and formulae components. Otherwise, the active components and pharmacological effects would result in differences and confusion. Therefore, the factors that would decrease the quality of research were excluded.

In addition, though the prevention and treatment efficacy of CMs on RIPI were promising, little details of ethical protocols were described in the reviewed studies. This is a very important aspect for patient safety in clinical trials. With regards to publications, 191 out of 199 were excluded due to the low quality of the studies, including 20 articles with neither any ethical approval nor patients’ agreement or signing informed consents. Only three clinical studies investigated the VC, FVC, and FEV1 in the treatment cases, the most important indices for pulmonary function.

Another critical issue of CMs is their toxicity and side effects. Herbal medicines have attracted more attention since aristolochic acid-induced nephropathy was reported (Cosyns, 2003). Though there was no toxicity reported in this review, some CMs have been reported to induce irritation (Zhu et al., 2012; Zhong et al., 2006) and be toxic in the endocrine system (Arbo et al., 2009), reproductive system (Tian et al., 2009), liver (Bilgi et al., 2010; Zhang et al., 2009), digestive system, nervous system (Xu et al., 2018; Chaouali et al., 2013), etc. The potential toxic CMs are listed in **Table 2**, in which herb-induced liver injury (HILI) can be assessed using the CIOMS/RUCAM scale (Fu et al., 2018). Furthermore, according to the Chinese Pharmacopeia, Armeniacae semen amarum was identified as “slightly toxic” (**Table 2**). Notably, this also includes that they would be “very toxic,” “toxic,” and “slightly toxic” if they had been used irrationally. Unfortunately, the maximal tolerance and LD50 are not standardized for many CMs as in Western medicine. Regarding this study, the papers mentioned the safety of CMs in which there were no particular methods, designs, and further index results for an LD50 investigation. Nevertheless, we still would like to sensitize the medical staff to the toxicity of CMs.

## Conclusion

In conclusion, although CMs might play a promising role in RIPI prevention and treatment, there is still a long way to go for CMs on RIPI. Further high-quality investigations in species, pharmacological effects and underlying mechanisms, and ethical and toxicological aspects are warranted.

## Author Contributions

XBW, XHW, and FC designed the study. YD, YCL, HL, and MLiu retrieved the literature. YD and YCL screened and double-checked the literature; YD and XBW analyzed the data and wrote the manuscript. MLi and YL polished the manuscript. All authors read and approved the final version.

## Funding

The study was financially supported by the National Natural Science Foundation of China (81874356); the Hubei Provincial Natural Science Foundation (2018CFC874); the Young Scientist Innovation Team Project of Hubei Colleges (T201510); the Hubei Province Health and Family Planning Scientific Research Project (WJ2017Z023); the Open Project of Hubei Key Laboratory of Wudang Local Chinese Medicine Research, Hubei University of Medicine (WDCM2018002); the Key Discipline Project of Hubei University of Medicine and the Foundation for Innovative Research Team of Hubei University of Medicine (2014CXG03, 2018YHKT01); and the Key Discipline Project of Hubei Province (2014XKJSXJ18).

## Conflict of Interest Statement

The authors declare that the research was conducted in the absence of any commercial or financial relationships that could be construed as a potential conflict of interest.
